# Isolation and Characterization of a *Phaseolus vulgaris* Trypsin Inhibitor with Antiproliferative Activity on Leukemia and Lymphoma Cells

**DOI:** 10.3390/molecules22010187

**Published:** 2017-01-23

**Authors:** Miao Li, Qin Liu, Yajuan Cui, Dong Li, Hexiang Wang, Tzi Bun Ng

**Affiliations:** 1 State Key Laboratory for Agrobiotechnology and Department of Microbiology, China Agricultural University, Beijing 100193, China; shuiyuemiaomiao@163.com; 2 Engineering Research Center of System-Nutrition, Beijing Research Institute for Nutritional Resources, Beijing 100069, China; 15801453639@163.com (Y.C.); xingyunxing_127@163.com (D.L.); 3 Institute of Plant Nutrition, Agricultural Resources and Environmental Science, Henan Academy of Agricultural Sciences, Zhengzhou 450002, China; liuqin_bio@hotmail.com; 4 School of Biomedical Sciences, Faculty of Medicine, The Chinese University of Hong Kong, Shatin, New Territories, Hong Kong, China; tzibunng@cuhk.edu.hk

**Keywords:** leguminous seeds, leukemia L1210, lymphoma MBL2, purification

## Abstract

A 17.5-kDa trypsin inhibitor was purified from *Phaseolus vulgaris* cv. “gold bean” with an isolation protocol including ion exchange chromatography on DEAE-cellulose (Diethylaminoethyl-cellulose), affinity chromatography on Affi-gel blue gel, ion exchange chromatography on SP-sepharose (Sulfopropyl-sepharose), and gel filtration by FPLC (Fast protein liquid chromatography) on Superdex 75. It dose-dependently inhibited trypsin with an IC_50_ value of 0.4 μM, and this activity was reduced in the presence of dithiothreitol in a dose- and time-dependent manner, signifying the importance of the disulfide linkage to the activity. It inhibited [methyl-^3^H] thymidine incorporation by leukemia L1210 cells and lymphoma MBL2 cells with an IC_50_ value of 2.3 μM and 2.5 μM, respectively. The inhibitor had no effect on fungal growth and the activities of various viral enzymes when tested up to 100 μM.

## 1. Introduction

Protease inhibitors have been isolated from the seeds of different monocots and dicots, including maize [1], wheat [2], wampee [3], bitter gourd [4], *Momordica cochinchinensis* [5], and legumes [6,7,8]. Some of them display a variety of biological activities including antifungal [9], immunomodulatory [5] and antitumor/antiproliferative [8,10] activities. Thus, they have drawn the attention of many researchers.

One class of protease inhibitors is trypsin inhibitors. Trypsin inhibitors are divided into Kunitz type [11], Bowman-Birk type [12,13], and squash type [4]. The three types have a molecular mass of about 20 kDa, 8 kDa, and 3 kDa, respectively. The soybean produces both Kunitz and Bowman-Birk inhibitors [6], while melons belonging to family Cucurbitaceal produce squash-type inhibitors [4].

Leguminous seeds are a rich source of proteins, including protease inhibitors, lectins [14], antifungal proteins [15], ribosome inactivating proteins [16], and arcelins [17]. Some of the proteins have interesting biological activities, such as insecticidal [14], immunomodulatory [5], antifungal [18], and antitumor [8,10] activities. The intent of the present study was to isolate a trypsin inhibitor from the gold bean and to test it for inhibitory action on tumor cells, viral enzymes, and fungal growth.

## 2. Results

### 2.1. Isolation of Trypsin Inhibitor

**Figure 1:**
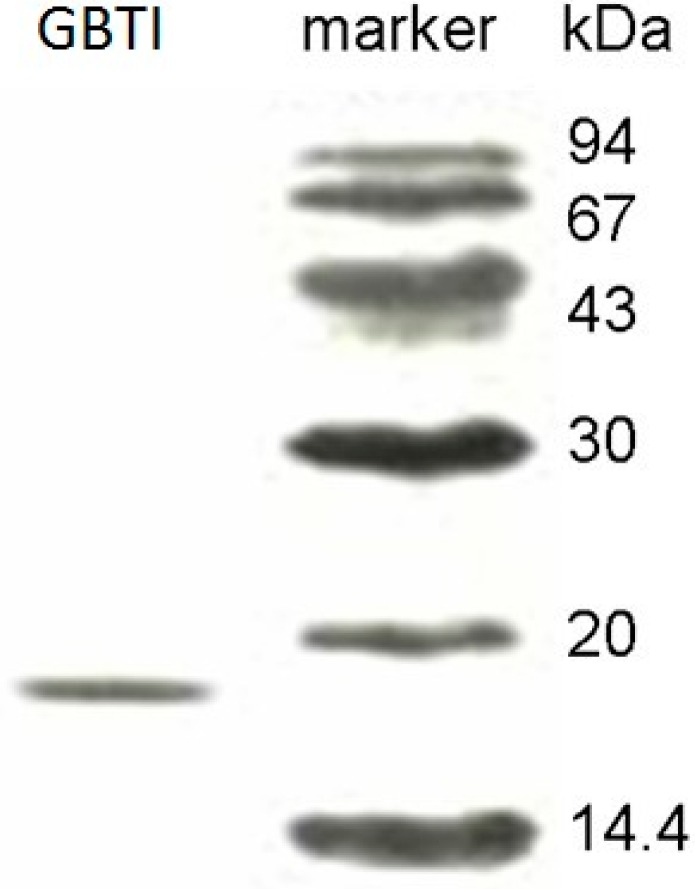
SDS-PAGE results of gold bean trypsin inhibitor (GBTI, which was fraction SU2, 12 μg) and molecular mass marker proteins from GE Healthcare. Molecular mass of trypsin inhibitor was 17.5 kDa. The marker proteins included phosphorylase b (94 kDa), bovine serum albumin (67 kDa), ovalbumin (43 kDa), carbonic anhydrase (30 kDa), soybean trypsin inhibitor (20 kDa), and α-lactalbumin (14.4 kDa).

Upon ion exchange chromatography on DEAE-cellulose, the bean extract was separated into three fractions of approximately equal size, an unadsorbed fraction D1 and two adsorbed fractions D2 and D3. Trypsin inhibitory activity was located in fraction D2. When fraction D2 was subjected to affinity chromatography on Affi-gel blue gel, the activity was recovered in the larger unadsorbed fraction B1 (data not shown). Upon ion exchange chromatography on SP-sepharose, fraction B1 was resolved into a small unadsorbed fraction S1 and two large adsorbed fractions (S2 and S3) of approximately equal size. Activity resided in fraction S2. Further purification of S2 on Superdex 75 yielded two fractions, SU1 and SU2. Only fraction SU2 exhibited trypsin inhibitory activity. SU2 was the purified trypsin inhibitor (GBTI). The yields of the various chromatographic fractions are presented in Table 1. The N-terminal sequence of the trypsin inhibitor is shown in Table 2. GBTI exhibited a molecular mass of 17.5 kDa in both sodium dodecyl sulfate-polyacrylamide gel electrophoresis (SDS-PAGE) and gel filtration (Figure 1). Its trypsin inhibitor activity remained unchanged in the temperature range 20–80 °C, was reduced by 60% at 90 °C, and, was totally abolished after exposure to 100 °C.

### 2.2. Biological Activities of Isolated Trypsin Inhibitor

**Figure 2:**
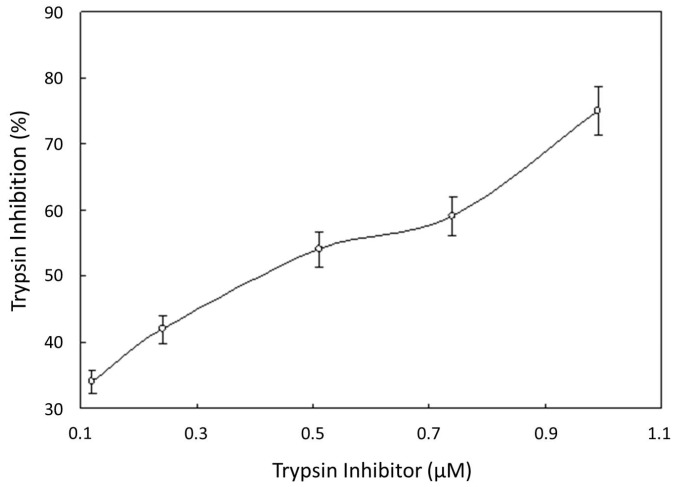
Inhibition of trypsin by **GBTI**. IC_50_ = 0.4 μM. Results are presented as mean ± SD (*n* = 3).

GBTI inhibited trypsin with an IC_50_ of 0.4 μM (Figure 2). Dithiothreitol (DTT) treatment lowered the trypsin inhibitory activity in a dose- and time-dependent manner (Table 3). The IC_50_ values of the inhibitory effects of GBTI on L1210 cells and MBL2 cells were 2.3 μM and 2.5 μM, respectively (Table 4). There was no inhibition on HIV-1 reverse transcriptase when it was tested at various concentrations up to 100 μM (Table 5). The inhibitor had no effect on the activities of HIV-1 integrase (Table 5) and SARS coronavirus proteinase at 100 μM. There was no inhibitory action on fungal growth at 100 μM (Table 6).

## 3. Discussion

Gold bean trypsin inhibitor (GBTI) is unadsorbed on Affi-gel blue gel, but adsorbed on DEAE-cellulose. This chromatographic behavior is distinctly different from that of other leguminous antifungal proteins which are unadsorbed on the ion exchanger and adsorbed on the affinity chromatography media [15,19]. Hence, the purification procedure described herein provides a convenient means to separate trypsin inhibitors from antifungal proteins which may be present in the same legume.

The trypsin inhibitor from gold beans demonstrate some sequence resemblance to other leguminous trypsin inhibitors including those of cowpea, mung bean, and garden pea. It is noteworthy that it exerts a potent antiproliferative action on both L1210 cells and MBL2 cells. Some of the legume trypsin inhibitors, for instance, field bean trypsin inhibitors, inhibit in vitro proliferation of cancer cells and metastasis in vivo [20,21]. Gold bean trypsin inhibitor is dissimilar from broad bean trypsin inhibitor [9] in that the former lacks HIV-1 reverse transcriptase inhibitory activity. The lack of HIV-1 reverse transcriptase inhibitory activity in gold bean trypsin inhibitor is similar to the findings on lily bulb trypsin inhibitor [22]. Gold bean trypsin inhibitor is also devoid of any inhibitory effect on HIV-1 integrase and SARS coronavirus proteinase. The absence of antifungal activity in gold bean trypsin inhibitor is also in agreement with the observation on some trypsin inhibitors, such as lily bulb trypsin inhibitor [22]. The results demonstrate that HIV-1 reverse transcriptase inhibitory and antifungal activities of trypsin inhibitors have structural requirements different from those of trypsin inhibitory activity.

The importance of the disulfide linkage in gold bean trypsin inhibitor to its trypsin inhibitory activity is revealed by the ability of the reducing agent dithiothreitol to reduce the activity. This is reminiscent of the results on trypsin-chymotrypsin inhibitor from *Vigna mungo* seeds [23].

A lectin, an antifungal protein and a trypsin inhibitor can be isolated from the gold bean [24,25]. All three proteins can be regarded as defense proteins that protect the plant from pathogenic and predatory organisms. Previously, a trypsin inhibitors have been isolated from *Phaseolus vulgaris*. However, they have not been examined for biological activities other than trypsin inhibitory activity [26,27,28,29,30,31,32].

In conclusion, a new trypsin inhibitor was purified from gold bean. It dose-dependently inhibited trypsin, and this activity was reduced in the presence of dithiothreitol in a dose- and time-dependent manner, signifying the importance of the disulfide linkage to the activity. It inhibited [methyl-3H] thymidine incorporation by leukemia L1210 cells and lymphoma MBL2 cells.

## 4. Materials and Methods

### 4.1. Isolation of Trypsin Inhibitor

A water extract of gold beans *Phaseolus vulgaris* cv. “gold bean” from Mainland China (260 g) was made by homogenizing them in distilled water (6 mL/g). The homogenate was then centrifuged (14,000× *g* for 25 min at 4 °C). The supernatant was collected and loaded on a 5 × 20 cm column of DEAE-cellulose (Sigma, St. Louis, MI, USA) in 10 mM Tris-HCl buffer (pH 7.4). Following removal of unadsorbed proteins (fraction D1), the column was eluted sequentially with 0.2 M NaCl and 1 M NaCl in the Tris-HCl buffer. Fraction D2 eluted with 0.2 M NaCl was dialyzed and then chromatographed on a 5 × 15 cm column of Affi-gel blue gel (Bio-Rad, Woodinville, WA, USA) in 10 mM Tris-HCl buffer (pH 7.4). The unadsorbed proteins (fraction B1) were dialyzed against 10 mM NH_4_Ac buffer (pH 5) and applied on a 2.5 × 20 cm column of SP-sepharose (GE Healthcare, Uppsala, Sweden). After elution of unadsorbed proteins (fraction S1), the column was eluted with a 0–1 M NaCl concentration gradient in the NH_4_Ac buffer. The first adsorbed fraction (S2) was then subjected to gel filtration on a Superdex 75 HR 10/30 column (GE Healthcare) in 0.2 M NH_4_HCO_3_ buffer (pH 8.5). The second absorbance peak (SU2) represented purified trypsin inhibitor (GBTI).

### 4.2. Assay for Trypsin Inhibitory Activity

The assay for trypsin inhibitory activity was carried out by addition of test sample (20 μL) to 160 μL of a 1% casein solution in 0.1 M Tris-HCl buffer (pH 7.4). Trypsin (20 μL of a 0.5 mg/ml solution) was then added and the mixture was incubated at 37 °C for 15 min before 0.4 mL 5% trichloroacetic acid was added to terminate the reaction. After centrifugation, the absorbance of the supernatant, which reflects the amount of casein fragments, was measured at 280 nm [22].

### 4.3. Electrophoresis, Molecular Mass Determination, and N-Terminal Sequence Analysis

The purified trypsin inhibitor was subjected to sodium dodecyl sulfate-polyacrylamide gel electrophoresis (SDS-PAGE, Woodinville, WA, USA) for molecular mass determination in accordance with the procedure of Laemmli and Favre [33]. Gel filtration on an FPLC-Superdex 75 column (GE Healthcare, Uppsala, Sweden), which had been calibrated with molecular mass markers including phosphorylase b (94 kDa), bovine serum albumin (67 kDa), ovalbumin (43 kDa), carbonic anhydrase (30 kDa), soybean trypsin inhibitor (20 kDa) and α-lactalbumin (14.4 kDa) (GE Healthcare), was conducted to determine the molecular mass of the protein. The N-terminal sequence of the trypsin inhibitor was determined by using a Hewlett-Packard HP G1000A Edman degradation unit (Hewlett Packard Company, Palo Alto, CA, USA) and an HP 1000 HPLC System (Hewlett Packard Company, Palo Alto, CA, USA).

### 4.4. Effect of Heat Treatment

The purified trypsin inhibitor was incubated in a water bath at different temperatures from 20 °C to 100 °C for 15 min, then immediately chilled on ice. The reaction mixture was then subjected to the assay of trypsin inhibitory activity.

### 4.5. Effect of Dithiothreitol (DTT) on Trypsin Inhibitory Activity

The purified trypsin inhibitor (2.5 μM) was incubated with dithiothreitol (DTT) at the final concentration of 2.5, 10, and 40 mM for 5, 20, and 80 min at 37 °C, respectively. For comparison, soybean trypsin inhibitor purchased from Sigma Chemical Co., (St. Louis, MI, USA) (2.5 μM) was similarly treated. The reaction was terminated by adding iodoacetamide at twice the amount of thiol functions at each DTT concentration. The remaining trypsin inhibitor activity was measured at pH 7.4, as described above. The highest iodoacetamide concentration used in the test was devoid of any effect on the activity of trypsin and the trypsin inhibitory activity of purified trypsin inhibitor and soybean trypsin inhibitor [34].

### 4.6. Assay for Antiproliferative Activity toward Leukemia Cells and Lymphoma Cells

The antiproliferative activity of the purified protein was determined as follows. The cell lines L1210 (leukemia) and MBL2 (lymphoma) were purchased from American Type Culture Collection. The cell line was maintained in Dulbecco Modified Eagles’ Medium (DMEM) supplemented with 10% fetal bovine serum (FBS) and 100 mg/L streptomycin and 100 IU/mL penicillin at 37 °C in a humidified atmosphere of 5% CO_2_. Cells (1 × 10^4^) in their exponential growth phase were seeded into each well of a 96-well culture plate (Nunc, Roskilde, Denmark) and incubated for 3 h before addition of the trypsin inhibitor. Incubation was carried out for another 48 h. Radioactive precursor, 1 μCi ([^3^H-methyl]-thymidine, from GE Healthcare), was then added to each well and incubated for 6 hrs. The cultures were then harvested by a cell harvester. The incorporated radioactivity was determined by liquid scintillation counting [15].

### 4.7. Assay for HIV-1 Reverse Transcriptase Inhibitory Activity

The assay for HIV reverse transcriptase inhibitory activity was carried out in view of the report that trypsin inhibitors manifest this activity [35,36]. It was conducted according to instructions supplied with the assay kit from Boehringer Mannheim (Basel, Switzerland). The assay takes advantage of the ability of reverse transcriptase to synthesize DNA, starting from the template/primer hybrid poly (A) oligo (dT) 15. The digoxigenin- and biotin-labeled nucleotides in an optimized ratio are incorporated into one of the same DNA molecule, which is freshly synthesized by the reverse transcriptase (RT). The detection and quantification of synthesized DNA as a parameter for RT activity follows a sandwich ELISA protocol. Biotin-labeled DNA binds to the surface of microtiter plate modules that have been pre-coated with strepatavidin. In the next step, an antibody to digoxigenin, conjugated to peroxidase enzyme, binds to the digoxigenin-labeled DNA. In the final step, the peroxidase substrate is added. The peroxidase enzyme catalyzes the cleavage of the substrate, producing a colored reaction product. The absorbance of the sample at 405 nm can be determined using a microtiter plate (ELISA) reader and is directly correlated to the level of RT activity. A fixed amount (4–6 ng) of recombinant HIV-1 reverse transcriptase was used. The inhibitory activity of the protein was calculated as percent inhibition compared to a control without the protein [15]. The positive control used was kale antifungal peptide.

### 4.8. Assay of Ability to Inhibit HIV-1 Integrase

#### 4.8.1. Expression and Purification of Recombinant HIV-1 Integrase

The plasmid that expressed His-tagged wild-type HIV-1 integrase, pT7-7-His (Y|TX)-HIV-1-IN, was a generous gift from Professor S.A. Chow (School of Medicine, UCLA). To express the protein, a 1-liter culture of *E. coli* BL21 (DE3) cells containing the expression plasmid was grown at 37 °C until OD600 reached 0.7–0.8. Cells were induced by addition of 0.8 mM IPTG (isopropyl-β-d-thiogalactopyranoside) and harvested, after 4 h of incubation, by centrifugation at 6000× *g* for 10 min at 4 °C Cells were suspended at a concentration of 0.1 g/mL wet cell paste in 20 mM Tris-HCl buffer (pH 8.0) containing 0.1 mM EDTA (Ethylenediamine Tetraacetic Acid), 2 mM β-mercaptoethanol, 0.5 M NaCl and 5 mM imidazole. Lysozyme was added to a concentration of 0.2 mg/mL. After incubation at 4 °C for 1 h, the lysate was sonicated and centrifuged at 40,000× *g* at 4 °C for 20 min. The pellet was homogenized in 50 mL buffer A (20 mM Tris-HCl, pH 8.0, 2 M NaCl, 2 mM β-mercaptoethanol) containing 5 mM imidazole. The suspension was rotated at 4 °C for 1 h, and cleared by centrifugation at 40,000× *g* at 4 °C for 20 min. The supernatant was loaded onto a 1-mL chelating Sepharose column (GE Healthcare) charged with 50 mM NiSO_4_ and was equilibrated with buffer A containing 50 mM imidazole. The column was washed with five column volumes of buffer A containing 5 mM imidazole, and the protein was eluted with three column volumes of buffer A containing 200 and 400 mM imidazole, respectively. Protein containing fractions were pooled, and EDTA was added to a final concentration of 5 mM. The protein was dialyzed against buffer B (20 mM HEPES, pH 7.5, 1 mM EDTA, 1 M NaCl, 20% glycerol) containing 2 mM β-mercaptoethanol, and then against buffer B containing 1 mM dithiothreitol. Aliquots of the protein were stored at −70 °C [19].

#### 4.8.2. HIV-1 Integrase Assay

A non-radioactive ELISA-based HIV-1 integrase assay was performed according to the DNA-coated plate method. In this study, 1 μg of Smal-linearized pBluescript SK was coated onto each well in the presence of 2 M NaCl as target DNA. The donor DNA was prepared by annealing VU5BR (5’-biotin-GTGTGGAAAATCTCTAGCAGT-3’) and VU5 (5’-ACTGCTAGAGATTTTCCACAC-3’) in 10 mM Tris-HC1, pH 8.0, 1 mM EDTA and 0.1 M NaCl at 80 °C, followed by 30 min at room temperature. Integrase reaction was performed in 20 mM HEPES (4-(2-Hydroxyethyl)-1-piperazineethanesulfonic Acid) (pH 7.5) containing 10 mM MnCl_2_, 30 mM NaCl, 10 mM dithiothreitol and 0.05% Nonidet-P40 (Sigma). After the integrase reaction, the biotinylated DNA immobilized on the wells was detected by incubation with streptavidin-conjugated alkaline phosphatase (Boehringer-Mannheim, Mannheim, Germany), followed by colorimetric detection with 1 mg/mL *p*-nitrophenyl phosphate in 10% diethanolamine buffer (pH 9.8) containing 0.5 mM MgCl_2_. The absorbance due to the alkaline phosphatase reaction was measured at 415 nm. The ribosome inactivating protein luffin was used as a positive control [16].

### 4.9. Screening for Inhibitory Effect on SARS Coronavirus Protease

The activity of SARS coronavirus (CoV) protease was indicated by a cleavage of designed substrate which was composed of two proteins linked by a cleavage site for SARS CoV protease. The reaction was performed in a mixture containing 5 μM SARS CoV protease, 5 μM sample, 20 μM substrate, and buffer [20 mM Tris-HCl (pH 7.5), 20 mM NaCl and 10 mM beta-mercaptoethanol] for 40 min at 37 °C. After 40 min, the reaction was stopped by heating at 100 °C for 2 min. Then the reaction mixture was analysed by SDS-PAGE. If SARS CoV protease is inhibited by the test sample, there is only one band, which is the intact substrate, shown in SDS-PAGE [19].

### 4.10. Assay of Antifungal Activity

This assay was conducted in view of the report on antifungal activity of some trypsin inhibitors [9]. The assay for antifungal activity toward *Mycosphaerella arachidicola* and *Fusarium oxysporum* was carried out in 100 mm × 15 mm Petri dishes containing 10 ml of potato dextrose agar. After the mycelial colony had developed, sterile blank paper disks (0.625 cm in diameter) were placed at a distance of 0.5 cm away from the rim of the mycelial colony. An aliquot (15 μL) of the purified trypsin inhibitor was added to a disk. The plates were incubated at 23 °C for 72 h until mycelial growth had enveloped the disks containing the control and had formed crescents of inhibition around the disks containing samples with antifungal activity [15]. The positive control used was kale antifungal peptide.

## 5. Conclusions

A 17.5-kDa trypsin inhibitor was purified from *Phaseolus vulgaris* cv. “gold bean”. It was adsorbed on DEAE-cellulose, unadsorbed on Affi-gel blue gel, and adsorbed on SP-sepharose. It dose-dependently inhibited trypsin with an IC_50_ value of 0.4 μM. About 80% and all of the trypsin inhibitory activity were abolished after treatment with 2.5 mM dithiothrietol for 5 min and 20 min of incubation, respectively. [Methyl-3H] thymidine incorporation by leukemia L1210 cells and lymphoma MBL2 cells was inhibited with an IC_50_ value of about 2 μM. Mycelial growth in *Fusarium oxysporum* and *Mycosphaerella arachidicola* were unaffected. The activities of HIV-1 reverse transcriptase, HIV-1 integrase and SARS coronavirus proteinase were unaltered after exposure to the trypsin inhibitor.

## Figures and Tables

**Table 1:** Yields and trypsin inhibitory activities of various chromatographic fractions (from 260 g dry gold beans).

Fraction	Yield (mg)	IC_50_ (μg/mL)	Purification Fold
**Extract**	**3140 ± 102**	**142.9 ± 5.1**	**1**
D1	730 ± 28	-	-
**D2**	**665 ± 35**	**40.2 ± 2.3**	**3.6 ± 0.3**
D3	836 ± 45	-	-
**B1**	**372.6 ± 38.1**	**28.5 ± 1.6**	**5.0 ± 0.6**
B2	124.2 ± 8.6	-	-
S1	53.9 ± 3.8	-	-
**S2**	**121.4 ± 7.3**	**11.3 ± 0.5**	**12.6 ± 1.1**
S3	94.5 ± 4.7	-	-
SU1	20.8 ± 1.2	-	-
**GBTI**	**64.7 ± 3.3**	**7.0 ± 0.4**	**20.4 ± 1.9**

Trypsin inhibitor-enriched fractions are highlighted in boldface. Results represent mean ± SD, *n* = 2.

**Table 2:** N-terminal sequence comparison of gold bean trypsin inhibitor with those from other leguminous species (identical corresponding amino acid residues are underscored).

Trypsin Inhibitor	Amino Acid Sequence																																							
GBTI (1–40)	H	N	D	S	L	D	E	P	S	E	S	V	E	P	N	L	D	I	Q	V	C	T	A	S	I	P	P	G	C	Q	Q	T	D	V	N	L	L	N	C	H
VRTI (29–68)	H	H	D	S	S	D	E	P	S	E	S	S	E	P	C	C	D	S	C	R	C	T	K	S	I	P	P	Q	C	H	C	A	D	I	R	L	N	S	C	H
VUTI (83–122)	D	S	D	S	S	D	E	P	S	E	S	S	E	P	C	C	D	S	C	I	C	S	K	S	I	P	P	Q	C	H	H	T	D	I	R	L	N	S	C	H
PSTI (42–81)	N	G	D	A	G	Y	S	I	K	S	T	T	T	A	C	C	D	S	C	I	C	T	K	S	I	P	P	Q	C	H	C	T	D	V	G	K	T	C	H	S

GBTI = gold bean trypsin inhibitor. GBTI (1–40) refers to the sequence of GBTI starting at amino acid residue #1 and ending at residue #40. VRTI = *Vigna radiata* var. radiata trypsin inhibitor. VRTI (29–68) refers to ythe sequence of VRTI starting at amino acid residue #29 and ending at residue #68. VUTI = *Vigna unguiculata* trypsin inhibitor. VUTI (83–122) refers to the sequence of VUTI starting at amino acid residue #83 and ending at residue #122. PSTI = *Phaseolus vulgaris* trypsin inhibitor. PSTI (42–81) refers to the sequence of PSTI starting at amino acid residue #42 and ending at residue #81. Amino acids identical to those of GBTI are underscored.

**Table 3:** Inhibition rate (%) of dithiothreitol (DTT) on the activity of GBTI and soybean trypsin inhibitor after incubation at 37 °C for different durations.

	GBTI			Soybean Trypsin Inhibitor
Incubation Time (min)	2.5 mM DTT	10 mM DTT	40 mM DTT	2.5 mM DTT
5	4.9 ± 0.6 ^a^	10.7 ± 1.2 ^b^	15.0 ± 1.5 ^c^	78.2 ± 5.7 ^a^
20	17.3 ± 1.9 ^d^	23.4 ± 2.3 ^e^	31.8 ± 2.7 ^f^	93.7 ± 5.0 ^b^
80	46.2 ± 4.1 ^g^	56.5 ± 4.7 ^h^	71.7 ± 5.6 ^i^	96.4 ± 4.3 ^c^

Results are presented as mean ± SD (*n* = 3). Different letters (e.g., ^a,b^ and ^c^) indicate statistically significant differences (*p* < 0.05) when (I) data at the same time point and different DTT concentrations or (II) data at the same DTT concentration, but different time points, were analyzed by analysis of variance followed by Duncan’s multiple range test. Inhibition rate (%) of purified or soybean trypsin inhibitor at x mM DTT = (Trypsin inhibitory activity of purified or soybean trypsin inhibitor−trypsin inhibitory activity of purified or soybean trypsin inhibitor in presence of x mM DTT)/trypsin inhibitory activity of purified or soybean trypsin inhibitor × 100%.

**Table 4:** Inhibition rate (%) of GBTI on L1210 cells and MBL2 cells.

Dose (μM)	L1210 Cells	MBL2 Cells
20	94.9 ± 6.2 ^a^	94.1 ± 6.3 ^a^
10	88.0 ± 5.8 ^a^	87.3 ± 5.9 ^a^
5	73.6 ± 5.2 ^b^	71.2 ± 6.1 ^b^
2.5	52.3 ± 4.0 ^c^	49.8 ± 4.7 ^c^
1.25	27.1 ± 3.2 ^d^	24.0 ± 3.1 ^d^
IC_50_ (μM)	2.3	2.5

Results are presented as mean ± SD (*n* = 3). Different letters (e.g., ^a,b,c^ and ^d^) indicate statistically-significant differences (*p* < 0.05) when data were analyzed by analysis of variance followed by Duncan’s multiple range test.

**Table 5:** Assay of GBTI for inhibitory activity toward HIV–1 integrase (In) and reverse transcriptase (RT).

% Inhibition of HIV-1 In Activity			% Inhibition of HIV-1 RT Activity		
Protein Concentration (μM)	Luffin	GBTI	Protein Concentration (μM)	Kale Antifungal Peptide	GBTI
0.35	19.2 ± 2.1	ND	0.1	12 ± 1.7	ND
0.7	78.3 ± 6.4	ND	0.32	22 ± 1.8	ND
1.4	96.5 ± 7.3	ND	0.65	41 ± 3.6	ND
10	ND	0.3 ± 0.2	1	68 ± 4.9	2.6 ± 0.3
25	ND	0.6 ± 0.3	1.3	79 ± 3.7	ND
100	ND	1.3 ± 0.4	10	98 ± 3.6	3.1 ± 1.6
			100	ND	1.6 ± 0.8

ND = not determined. Results are means ± SD (*n* = 3).

**Table 6:** Assay of GBTI for antifungal activity toward *Mycosphaerella arachidicola* and *Fusaruim oxysporum.*

	*Mycosphaerella arachidicola* Colony Diameter (mm)		*Fusaruim oxysporum* Colony Diameter (mm)	
Concentration of Antifungal Peptide or Trypsin Inhibitor (μM)	Kale Antifungal Peptide	GBTI	Kale Antifungal Peptide	GBTI
0	25.2 ± 1.6	25.2 ± 1.6	28.3 ± 0.8	28.3 ± 0.8
0.4	20.7 ± 1.1	26.4 ± 1.3	24.1 ± 1.5	29.1 ± 1.1
2	14.1 ± 1.0	25.8 ± 1.4	18.4 ± 0.9	28.9 ± 0.9
10	9.3 ± 0.7	26.8 ± 0.9	10.2 ± 0.8	27.2 ± 0.7
50	ND	25.9 ± 1.0	ND	26.5 ± 0.9
100	ND	24.7 ± 1.9	ND	29.3 ± 1.2

ND = not determined. Results are means ± SD (*n* = 3).
